# An Experimental Study on Combustion and Cycle-by-Cycle Variations of an N-Butanol Engine with Hydrogen Direct Injection under Lean Burn Conditions

**DOI:** 10.3390/s22031229

**Published:** 2022-02-06

**Authors:** Weiwei Shang, Xiumin Yu, Weibo Shi, Zhao Chen, Huiying Liu, He Yu, Xiaoxue Xing, Tingfa Xu

**Affiliations:** 1 State Key Laboratory of Automotive Simulation and Control, Jilin University, Renmin Street 5988, Changchun 130012, China; yuxm@jlu.edu.cn (X.Y.); wbshi@jlu.edu.cn (W.S.); zhaochen12@mail.jlu.edu.cn (Z.C.); 2 Electronic Information Engineering College, Changchun University, Renmin Street 6543, Changchun 130022, China; huiying20@mails.jlu.edu.cn (H.L.); yuh82@ccu.edu.cn (H.Y.); xingxx@ccu.edu.cn (X.X.); 3 School of Optics and Photonics, Beijing Institute of Technology, Beijing 100081, China; ciom_xtf1@bit.edu.cn

**Keywords:** hydrogen direct injection, cycle-by-cycle variations, lean burn conditions, SI n-butanol engine

## Abstract

This study experimentally investigated the effects of hydrogen direct injection on combustion and the cycle-by-cycle variations in a spark ignition n-butanol engine under lean burn conditions. For this purpose, a spark ignition engine installed with a hydrogen and n-butanol dual fuel injection system was specially developed. Experiments were conducted at four excess air ratios, four hydrogen fractions(φ(𝐻2)) and pure n-butanol. Engine speed and intake manifold absolute pressure (MAP) were kept at 1500 r/min and 43 kPa, respectively. The results indicate that the θ0–10 and θ10–90 decreased gradually with the increase in hydrogen fraction. Additionally, the indicated mean effective pressure (IMEP), the peak cylinder pressure (Pmax) and the maximum rate of pressure rise ((dP/dφ)max) increased gradually, while their cycle-by-cycle variations decreased with the increase in hydrogen fraction. In addition, the correlation between the (dP/dφ)max and its corresponding crank angle became weak with the increase in the excess air coefficient (λ), which tends to be strongly correlated with the increase in hydrogen fraction. The coefficient of variation of the Pmax and the IMEP increased with the increase in λ, while they decreased obviously after blending in the hydrogen under lean burn conditions. Furthermore, when λ was 1.0, a 5% hydrogen fraction improved the cycle-by-cycle variations most significantly. While a larger hydrogen fraction is needed to achieve the excellent combustion characteristics under lean burn conditions, hydrogen direct injection can promote combustion process and is beneficial for enhancing stable combustion and reducing the cycle-by-cycle variations.

## 1. Introduction

Energy crisis, environmental pollution and increasingly stringent emissions regulations have promoted interest in alternative fuel sources. Thus, clean, green and renewable alternative fuels have become a research hotspot in the field of the internal combustion engine [1,2,3]. At the same time, high efficiency and low pollution engine technology, such as composite injection, is equally important. Combining alternative fuels with new technologies to improve engine combustion and emissions performance has great research significance and application prospects [4,5].

There are many kinds of alternative fuels for internal combustion engines, which can be divided into gas fuels and liquid fuels according to their forms. The gaseous alternative fuels mainly include natural gas (liquefied petroleum gas), hydrogen gas, propane, etc., while the liquid alternative fuels mainly include alcohols, biomass fuels, dimethyl ether (DME), etc. [6].

Among the many alternative fuels, renewable alcohol fuels have drawn much attention as alternative pure fuels, as well as fuel blends for SI engines, such as methanol, ethanol and n-butanol. The properties of alcohol fuels are listed in Table 1 [7,8]. Compared to traditional fuel gasoline, the use of methanol and ethanol in alcohol fuels has the disadvantages of a low energy content, being strongly corrosive to distribution pipes and a reduced compatibility with existing power equipment. In contrast, n-butanol has several advantages over methanol and ethanol. N-butanol has the most similar physical and chemical properties to gasoline and is considered as a promising alternative fuel for gasoline [6]. N-butanol has higher energy density, lower latent heat of evaporation and lower corrosivity, which make it compatible with existing fuel systems and allows its use in SI power units. In addition, n-butanol fuel engines have a greater coefficient of fuel utilization and engine cold start performance due to the higher energy density and lower latent heat of evaporation of n-butanol. In addition, with the development of n-butanol production technology, the production cost of n-butanol has gradually reduced. N-butanol is prepared from a wide range of raw materials with low prices, mainly wheat, corn, pulp waste liquid, molasses and wild plants [9,10,11,12]. These characteristics make n-butanol more advantageous than methanol and ethanol as an alternative fuel for internal combustion engines and it has become one of the most promising alternative fuels for internal combustion engines.

At present, many researchers have studied the use of n-butanol as an engine fuel. In SI engines, most studies have focused on n-butanol that is blended with other fuels. There have been many studies on improving the performance of gasoline engines by using n-butanol as a small amount of blending fuel [13,14,15,16]. Balaji Dhanapal et al. [17] studied the effects of ethanol and isobutanol concentrations on engine performance, combustion and emissions in a spark ignition single-cylinder four-stroke engine. The results showed that isobutanol and ethanol could improve the engine’s operating parameters and decreased the gas emissions produced. Abdulfatah et al. [18] acquired and analyzed n-BH16 (8% n-butanol, 84% gasoline and 8% hydrogen) in a modified turbocharged GDI engine as a feasible alternative to neat fossil fuel. They found that particles emitted with n-BH16 decreased. A large number of studies have shown that the use of butanol as a partially blended fuel can effectively improve the performance of gasoline engines. Yu et al. [19] investigated the emission and combustion characteristics of an SI engine fed with gasoline and n-butanol blends under direct injection. They found that the addition of 20% n-butanol to the blends could reduce both particle and gaseous emissions. Wang et al. [20] explored the combustion and particle emission characteristics of gasoline blended with n-butanol under different injection methods and conditions. Their results showed that the dual injection of n-butanol and gasoline could decrease particle number and particle matter effectively.

There is still little research on pure butanol engines. Sandhu et al. [21] analyzed the combustion and emission characteristics of neat n-butanol using a single-cylinder SI engine with fuel port injection. The results showed that n-butanol had similar combustion characteristics to those of gasoline. However, n-butanol engines yield lower NOx, unburnt hydrocarbons and carbon dioxide emissions. Liang et al. [22] proposed n-butanol as a fuel for spark ignition aviation piston engines to replace gasoline. The engine performance when fueled with n-butanol was studied and compared to the use of gasoline. Their study showed that, compared to gasoline, n-butanol engines have a shorter flame development time, faster combustion time, more concentrated heat release, higher braking thermal efficiency and significantly lower CO and NOx emissions. However, the application of n-butanol l resulted in a significant increase in fuel consumption. Wang et al. [23] used an intelligent regression model to predict the cyclic variation, aiming to comparatively investigate the influence of operation parameters on the cyclic variation of the Wankel rotary engine with gasoline and n-butanol as the main fuels. Their results showed that, at the same conditions, the cyclic variation of speed with gasoline as the main fuel was lower than that with n-butanol.

Although n-butanol has proved to be feasible as an engine fuel, pure n-butanol combustion in SI engines still has the problem of insufficient dynamic properties compared to gasoline [24]. Hydrogen, as a clean and renewable fuel, is considered as a promising fuel for internal combustion engines because of its excellent fuel characteristics, such as low ignition energy and fast flame propagation. However, the utilization of hydrogen as a pure fuel is constrained by high infrastructure costs, a low energy density and storage difficulties. Therefore, the optimal application is to have hydrogen as a small amount of blending fuel to improve engine performance. Now, many studies have confirmed that adding hydrogen to the engine can improve the combustion characteristics of SI engines and reduce emissions. Meng et al. [25] studied the combustion and emission characteristics of a hydrogen direct injection stratified n-butanol engine. They found that with hydrogen direct injection, the power and fuel economy performance of the n-butanol engine were significantly improved and harmful emissions were reduced. Su et al. [26,27,28] explored the influence of hydrogen supplements on enhancing the idle performance of an n-butanol rotary engine. The experimental results indicated that the increase in the volume percentage of hydrogen in the total intake could reduce the engine instability, fuel energy flow rate and HC and CO emissions. Hao Meng et al. [29] devoted themselves to improving the idling performance of n-butanol rotary engines by blending in hydrogen and reducing the idling speed. Their test results showed that the engines could achieve better stability and economy due to the combined effect of blending in hydrogen and reducing the idling speed. Additionally, the addition of hydrogen is an effective way to reduce the period of flame development and propagation and the emissions of CO and HC. Zhang et al. [30] experimentally studied the effect of hydrogen port injection on the further improvement of n-butanol engine performance under part load and lean conditions. Their experimental results showed that hydrogen addition could heighten the brake thermal efficiency and the lean burn limit and decrease the ignition delay, rapid combustion duration and CO and HC emissions. Shang et al. [31] simulated and analyzed the idling performance of four hydrogen fractions on an n-butanol SI engine with directly injected hydrogen. The simulation results showed that the hydrogen addition could increase the peak cylinder pressure, accelerate and concentrate the heat release process, reduce HC, CO and acetaldehyde emissions and improve the idle combustion performance of butanol engines.

In view of this previous research, the results have shown that the current research on hydrogen–n-butanol engines mainly focuses on hydrogen intake injection and that the experiments were carried out using only limited variables and under almost stoichiometric conditions [32,33,34]. In addition, existing studies have shown that the performance of n-butanol engines is not satisfactory under lean burn conditions [35,36,37]. Therefore, it is very important to explore the potential of hydrogen in improving the performance of n-butanol engines under lean burn conditions. As far as we know, there are few studies on SI engines with n-butanol as the main work fuel port injection and a small ratio of hydrogen direct injection. Inspired by this, we experimentally studied the influence of hydrogen direct injection on combustion and the cycle-by-cycle variation performance of an n-butanol engine at different excess air ratios and hydrogen fractions. The main contributions of this paper are as follows: (1) the pioneering combination of hydrogen direct injection and lean combustion technology was applied to butanol engines; (2) the potential of hydrogen direct injection in optimizing n-butanol engine performance was explored; (3) some valuable and important information was provided regarding the combustion stability of n-butanol engines.

## 2. Experimental Apparatus and Procedures

### 2.1. Experimental Setup

**Figure 1:**
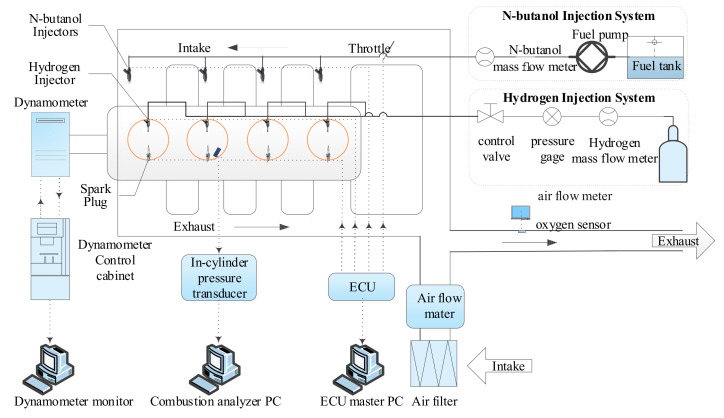
A schematic diagram of the test engine.

The research was carried out on a four-cylinder four-stroke modified dual injection SI engine. The main parameters of the engine are shown in Table 2. The test engine was a n-butanol–hydrogen composite injection engine with an independent hydrogen in-cylinder direct injection supply system and an n-butanol port injection supply system on the base of the original engine. A schematic diagram of the experimental setup is shown in Figure 1. We can see from the Figure 1 that the engine control parameters, such as injection duration, injection timing and the throttle opening, for the hydrogen and n-butanol were all accurately controlled by the electronic control unit (ECU).

The test equipment used in the experiment and its resolutions are shown in Table 3. In this experiment, a CW160 eddy current dynamometer was coupled with the test engine, which was used to control and measure the speed and output torque of the engine. The excess air ratio was obtained by an ETAS Lambda Meter 4 broadband oxygen sensor. A Kistler 2614B crank angle encoder was used to measure the cylinder pressure. An AVL-GU13Z-24 pressure transducer was mounted in the second cylinder and provided a charge signal, representing the pressure in the cylinder for the combustion analyzer. A Kistler 2614B crank angle encoder was used to obtain the crank angle. A DEWE soft combustion analyzer was used to collect and calculate all combustion data. The hydrogen fraction was defined as the heat energy ratio of the whole fuel. The hydrogen doping ratio in the test was not measured directly but was calculated by Formula 1 using the mass of the butanol and hydrogen, which were directly measured by an n-butanol consumption meter and hydrogen mass flow rate, respectively.

### 2.2. Experimental Methods

In this experiment, the injection mode was n-butanol port injection plus hydrogen direct injection, and the effects of the hydrogen direct injection on the combustion and cycle-by-cycle variations of the n-butanol engine under different excess air coefficients and hydrogen fractions were studied. The experiments were carried out at four excess air ratios (1, 1.1, 1.2, 1.3) and four hydrogen fractions (5%, 10%, 15%, 20%), and pure butanol was selected as the contrast test. During the experiments, the engine speed was fixed at 1500 r/min and the MAP was 43 kPa, so as to represent the typical working conditions in a city. The start of the hydrogen injection time was set as 100 °CA BTDC, and the hydrogen injection pressure was kept at 5 MPa. The ignition advance angle was set at the MBT.

### 2.3. Definition of Correlated Parameters

In this study, the excess air coefficient of the hydrogen and n-butanol mixture was defined as in Equation (1):

(1) $$\lambda =\frac{Q_{\mathrm{ma}}}{Q_{\mathrm{mh}}{\mathrm{AF}}_{h}+Q_{\mathrm{mn}}{\mathrm{AF}}_{n}}$$

where $Q_{\mathrm{ma}}$ denotes the air mass flow rate in kg/h; $Q_{\mathrm{mh}}$ and $Q_{\mathrm{mn}}$ represent the mass flows of hydrogen and n-butanol, respectively, in kg/h; ${\mathrm{AF}}_{h}\;$ and ${\mathrm{AF}}_{n}$ are the stoichiometric air fuel ratios of hydrogen and n-butanol (${\mathrm{AF}}_{h}$=34.3 and ${\mathrm{AF}}_{n}$ =14.6).

The hydrogen fraction was defined as the heat energy ratio of the whole fuel, which was defined as in Equation (2):

(2) $$\varphi \left(H_{2}\right)=\frac{M_{H_{2}}H_{\mu }\left(H_{2}\right)}{M_{H_{2}}H_{\mu }\left(H_{2}\right)+M_{N}H_{\mu }\left(N\right)}$$

where $M_{H_{2}}$ and $M_{N}$ represent the mass of hydrogen and n-butanol in each cycle, respectively, in kg; $H_{\mu }\left(H_{2}\right)$ and $H_{\mu }\left(N\right)$ denote the low calorific values of hydrogen and n-butanol, in kJ/kg.

The coefficient of the variation (COV) of the engine refers to the fluctuation in cylinder pressure during different cycles due to the difference in combustion state in the cylinder when the engine is running in a steady state, which is an important symbol of engine stability. The cyclic variation coefficient of the pressure related parameters (${\mathrm{COV}}_{x}$) is usually used to characterize the cyclic variation of engine combustion. The ${\mathrm{COV}}_{x}$ was defined as:

(3) $${\mathrm{COV}}_{x}=\frac{\sigma_{x}}{\bar{x}}\times 100\%$$

where

(4) $$\sigma_{x}=\sqrt{\frac{\sum_{i=1}^{N}{\left(x_{i}-\bar{x}\right)}^{2}}{N}}$$

(5) $$\bar{x}=\frac{1}{N}\overset{N}{\underset{i=1}{\sum }}x_{i}$$

where x represents a certain combustion performance parameter;

${\mathrm{COV}}_{x}$ represents the cyclic variation coefficient of x; $x_{i}$ is the combustion performance parameter of the i-th cycle of the engine; N indicates the sampled test cycles (the value of N in this experiment was 200); $\bar{x}$ represents the mean value of the characteristic parameter for a specific combustion.

## 3. Results and Analysis

### 3.1. MBT Spark Timings

**Figure 2:**
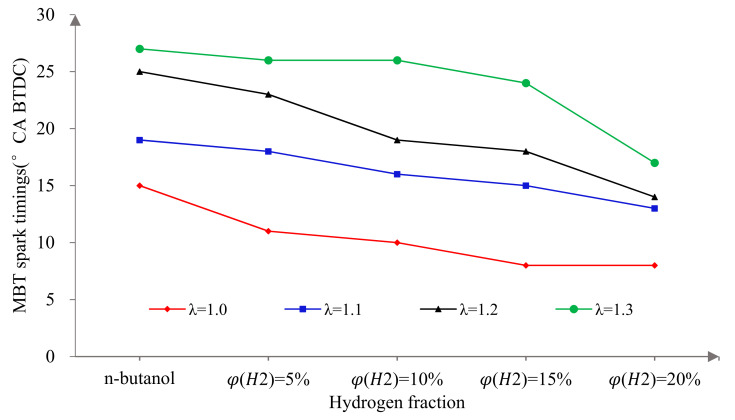
The MBT spark timings versus hydrogen fractions at different excess air ratios.

Figure 2 shows the variation of MBT spark timings with λ and φ(𝐻2). Firstly, it can be seen that the MBT spark timings decreased with the increase in φ(𝐻2). This is because the faster the laminar flame of propagates that hydrogen has, which makes the combustion faster and more concentrated. The forward combustion phase causes more fuel to burn before the compression TDC and the compression negative work increases significantly. Therefore, it was necessary to delay the ignition properly to obtain the appropriate combustion phase and make full use of the advantages of hydrogen direct injection in the cylinder to improve the power performance of the n-butanol engine. Secondly, the MBT spark timings increased with the increase in λ. The reason for this phenomenon is that the larger the λ, the thinner the mixture. The ignition stability was poor and the flame propagation speed was slow. So, on the one hand, the ignition advance angle should be increased to ensure the formation of a stratified combustion mode with a certain concentration of hydrogen enrichment near the spark plug in order to achieve stable ignition and flame propagation. On the other hand, the increase in λ results in slow combustion and an increase in post-combustion, which reduces the utilization rate of the fuel. Therefore, the ignition advance angle needed to be properly advanced in order to obtain the appropriate combustion phase.

### 3.2. Combustion Characteristics

**Figure 3:**
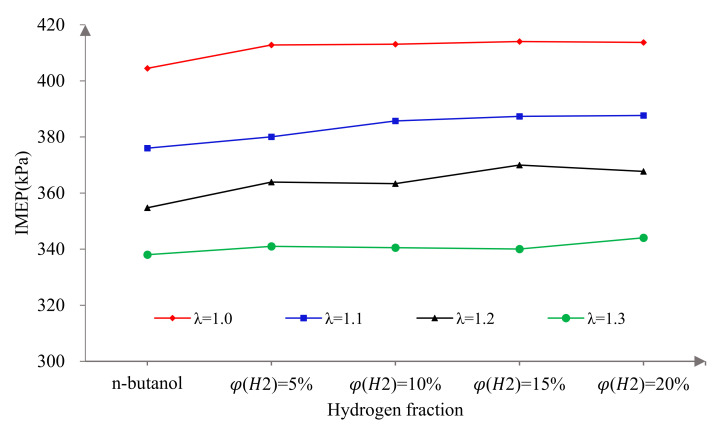
The effects of hydrogen fractions on IMEP at different excess air ratios.

Figure 3 shows the effects of φ(𝐻2) on the IMEP at different excess air ratios. From the vertical perspective, it can be seen that IMEP decreased monotonically with the increase in λ. This is mainly because the higher the λ, the thinner the mixture and the less the total amount of fuel per cycle under the condition of constant throttle opening, thus leading to the decrease in the IMEP. This is mainly because with the increase in λ, the mixture concentration decreases, resulting in a slower combustion speed and longer combustion duration, so the proportion of fuel burned before TDC increases and the power performance of the engine is weakened. In addition, within the test range, the larger λ is, the greater the improvement in IMEP by hydrogen addition. The effect of 20% hydrogen addition on increased the IMEP by 2.28%, 3.10%, 3.66% and 2.97%, corresponding to each λ. This is due to the excellent combustion characteristics of hydrogen, such as a wide ignition limit, low ignition energy and fast flame propagation. Hydrogen addition can shorten the combustion duration, maintain the concentrated combustion of fuel, improve the concentration of combustion and heat release and improve the power capacity of the engine under lean burn conditions.

Horizontally, the IMEP increased monotonically with the increase in φ(𝐻2) under the same conditions. This can be explained mainly by the fuel characteristics and injection mode. On the one hand, hydrogen direct injection forms a local hydrogen-rich region near the spark plug, which is conducive to reliable ignition. On the other hand, n-butanol has a large latent heat of evaporation, poor evaporation and atomization ability and a low laminar flame speed, so an n-butanol engine has the problem of ignition difficulty and slow initial flame development speed. However, hydrogen has good combustion characteristics, such as a low ignition energy and fast flame propagation and, as a gas fuel, hydrogen does not need to undergo the evaporation and atomization process. Therefore, hydrogen addition greatly improves the combustion speed of the mixture and makes up for the weakness of n-butanol fuel, making the combustion process more complete and then improving the power performance of the engine. When λ was 1.0, with the increase in φ(𝐻2), the increase in the IMEP showed a gradual decreasing tendency. In other words, the improvement in power performance by blending hydrogen is mainly reflected by whether hydrogen is added or not, and the sensitivity to φ(𝐻2) is small. This is because a small amount of hydrogen can ensure the stability of ignition and play a role in promoting flame propagation under stoichiometric conditions. Therefore, the sensitivity of φ(𝐻2) to combustion decreases. With the increase in λ, a larger hydrogen fraction was needed to achieve better results. This is because under lean burn conditions, the combustion environment was poor and more hydrogen was needed to ensure stable ignition and promote flame propagation.

**Figure 4:**
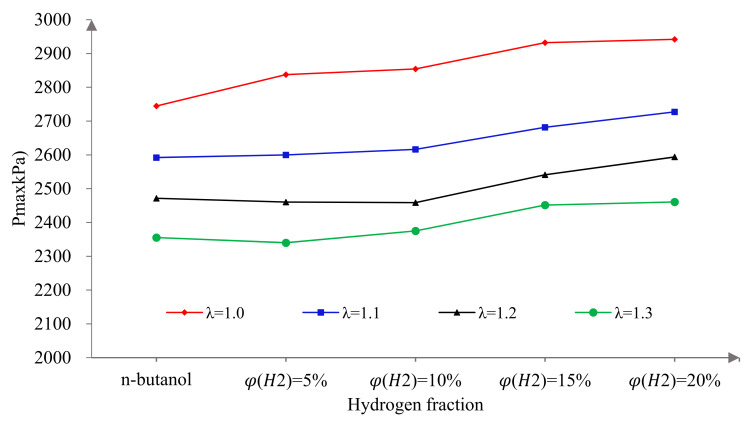
The effects of hydrogen fractions on Pmax at different excess air ratios.

Figure 4 shows that effects of hydrogen fractions on Pmax at different excess air ratios. It can be seen that the Pmax decreased gradually with the increase in λ. This is mainly because with the increase in λ, the mixture becomes thinner, the combustion speed slows down and the total amount of fuel per cycle also decreases, resulting in the decrease in cylinder pressure. With the increase in φ(𝐻2), the Pmax increased monotonically. Compared to pure n-butanol, the Pmax increased by 7.18%, 5.21%, 4.95% and 4.47% when φ(𝐻2) was 20% at λ 1.0, 1.1, 1.2 and 1.3, respectively. This is because hydrogen has the advantages of a wide ignition limit, low ignition energy and fast flame propagation speed, which effectively compensate for the defects of the large latent heat of evaporation and low saturated vapor pressure of n-butanol. As mentioned in the analysis of the influence of hydrogen on the IMEP, this is also attributed to the composite injection mode of the hydrogen and n-butanol combined with the excellent fuel characteristics of hydrogen. As can be seen from Figure 2, the MBT spark timings gradually advanced with the increase in φ(𝐻2). It is well known that the peak cylinder pressure increases with the increase in ignition advance angle [38]. Although at the MBT ignition timings, the cylinder pressure peak after the hydrogen addition still increased, compared to pure butanol. This indicates that hydrogen direct injection effectively improved the combustion process of the butanol engine. In addition, a small amount of hydrogen could significantly increase the Pmax at λ = 1.0, but a larger proportion of hydrogen was required under lean burn conditions. The reasons are the same as for the IMEP and will not be described here.

**Figure 5:**
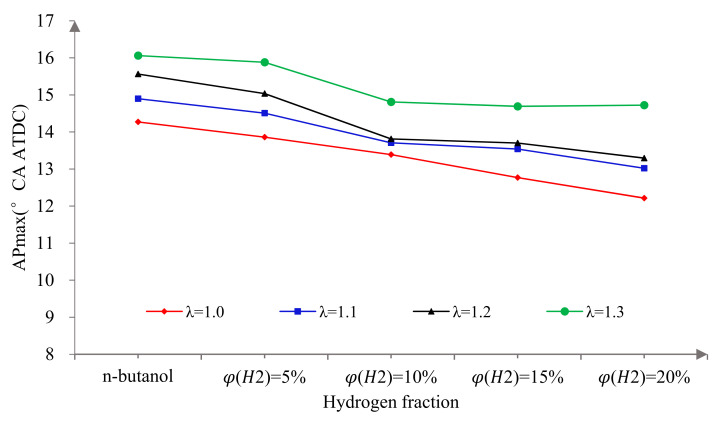
The effects of hydrogen fractions on APmax at different excess air ratios.

Figure 5 illustrates the effects of hydrogen fractions on APmax at different excess air ratios. As can be seen from the Figure 4, with the increase in φ(𝐻2), the APmax gradually moved forward and approached the top dead center. This is because hydrogen addition promotes the formation of a uniform mixture, improves the combustion speed and then advances the phase of the peak cylinder pressure.

**Figure 6:**
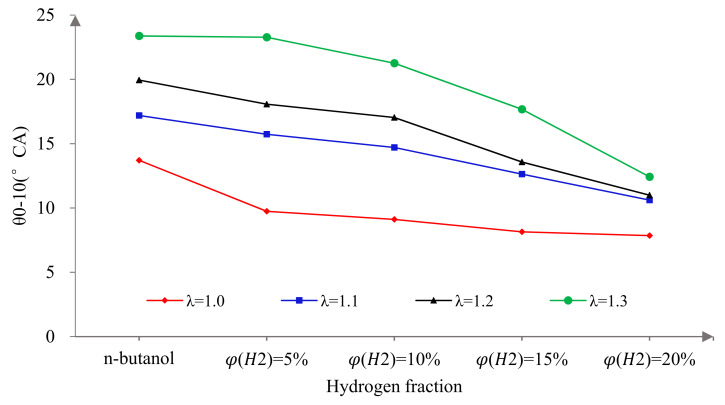
The effects of hydrogen fractions on θ0-10 at different excess air ratios.

**Figure 7:**
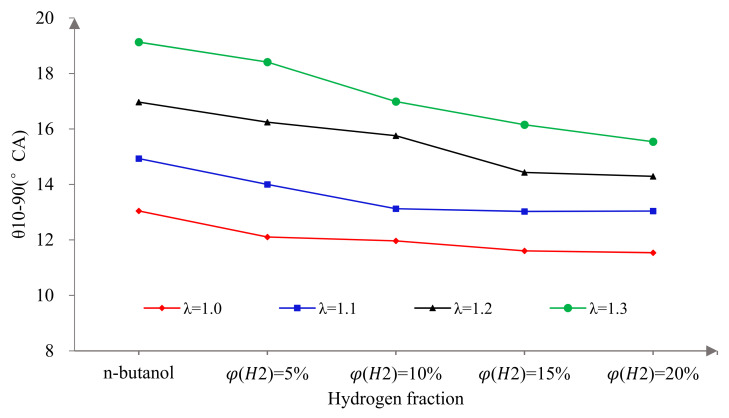
The effects of hydrogen fractions on θ10–90 at different excess air ratios.

CA0–10 is the duration of the crank angle from ignition to 10% heat release, which represents the early flame development and propagation. θ10–90 is the duration of the crank angle from 10% to 90% heat release, which represents the rapid combustion duration [39]. Figure 6 and Figure 7 show the effects of hydrogen fractions on θ0–10 and θ10–90 at different excess air ratios. It can be seen that the θ0–10 and θ10–90 were prolonged with the increase in λ. This is mainly because, with the increase in λ, the mixture becomes thinner, the ignition delay is prolonged and the flame propagation and diffusion speeds decrease, which results in the decrease in the combustion speed and increase in θ0–10 and θ10–90. Secondly, it is obvious that hydrogen addition could effectively shorten the θ0–10 and θ10–90. This is because hydrogen has many excellent fuel characteristics, which can effectively improve the problems of a slow combustion rate and long θ0–10 caused by a large latent heat of evaporation and low saturated vapor pressure. The specific analysis is as follows. First of all, the low ignition energy of hydrogen can guarantee the stability of ignition, which lays a foundation for initial combustion and effectively promotes the atomization process of n-butanol fuel. Secondly, hydrogen can enhance the mixture process due to its higher diffusion coefficient. Thirdly, hydrogen addition is beneficial to flame propagation at the initial stage of combustion and promotes the combustion in the cylinder due to the fast laminar flame speed. The θ0–10 of an n-butanol engine is shortened by blending in hydrogen. Hydrogen addition promotes early flame development and propagation and provides a higher cylinder pressure and temperature environment for the rapid combustion duration, thus promoting the heat release rate of the fuel in the subsequent combustion process and shortening the θ10–90. When λ = 1.0, the increase in φ(𝐻2) and the impact of the hydrogen addition on the θ0–10 and θ10–90 tended to decrease. This is because the improvement in combustion by blending in hydrogen is mainly seen during ignition and combustion by promoting flame propagation, and a small amount of hydrogen can achieve this goal under stoichiometric conditions. Therefore, the sensitivity of θ0–10 and θ10–90 to φ(𝐻2) decreased. While it can also be seen that the greater the λ, the more sensitive the θ0–10 and θ10–90 were to φ(𝐻2). This is because, under lean burn conditions, the pressure and temperature in the cylinder at initial combustion are lower, which is not conducive to the evaporation of n-butanol, so the mixture is more difficult to ignite. As a result, a larger hydrogen fraction was needed to achieve the excellent combustion characteristics.

### 3.3. Cycle-by-Cycle Variation

Cycle-by-cycle variation is one of the characteristics of SI engines and is an important characteristic for evaluating the combustion process and stability [40,41,42]. The parameters characterizing the cycle-by-cycle variation of engines can be generally divided into those related to cylinder pressure and those related to combustion rate. In this section, the parameters related to cylinder pressure were selected to analyze their cylinder pressure cycle distribution and their correlation. Considering the cyclic changes have similar regularity under lean burn conditions, λ was selected as 1.0 and 1.3 in this section, representing stoichiometry conditions and lean burn conditions, respectively.

#### 3.3.1. Cycle-by-Cycle Variation of Derived Pressure

**Figure 8:**
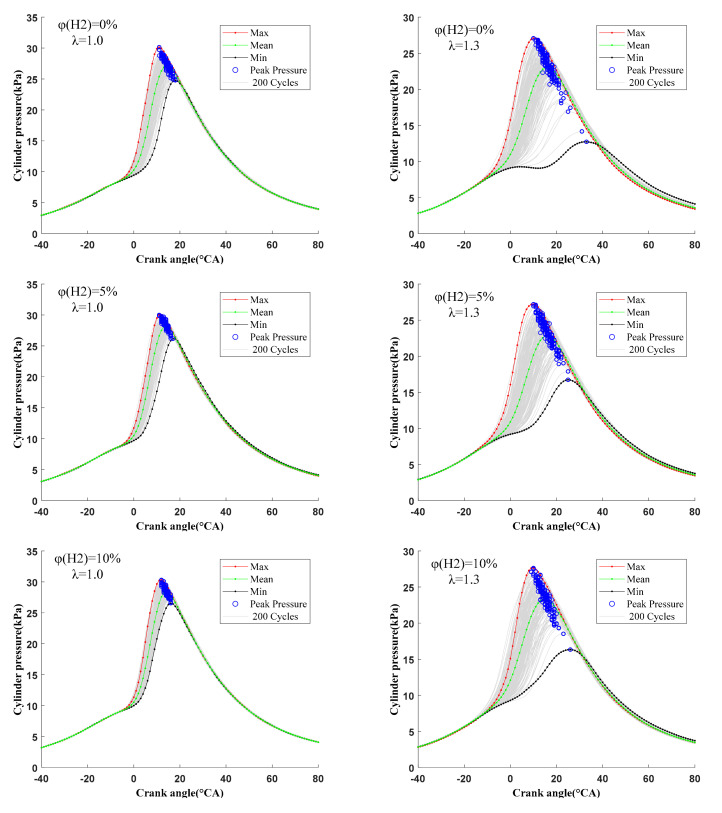

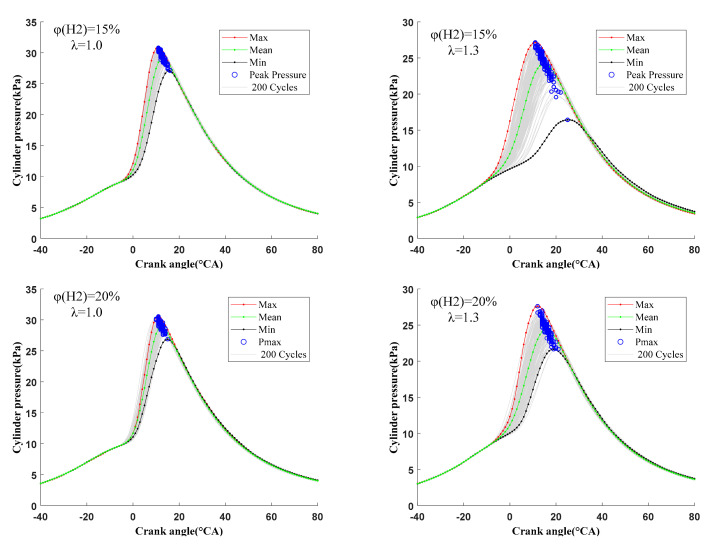
The cycle-by-cycle variation of cylinder pressure at different hydrogen fractions under λ = 1.0 and 1.3.

Figure 8 shows the curve of the cylinder pressure with the crank angle for 200 cycles under different hydrogen fractions and λ. The “Max”, “Mean” and “Min” represent the cylinder pressure curves corresponding to the maximum, average and minimum cylinder pressure peak values, respectively. The “blue circle” represents the peak cylinder pressure for 200 cycles. Through the influence of φ(𝐻2) on 200 cycles value of cylinder pressure collected continuously from the n-butanol engine with hydrogen direct injection, it can be seen that the hydrogen addition could significantly improve the cycle-by-cycle variation of cylinder pressure. First of all, with the increase in φ(𝐻2), the distribution of the cylinder pressure became more concentrated, i.e., the smaller the coefficient of variation. Secondly, with the increase in φ(𝐻2), the difference between the maximum and minimum cylinder pressure curves decreased, i.e., the maximum difference between cycles became smaller. Thirdly, with the increase in φ(𝐻2), the pressure value showed an increasing trend and the crank angle corresponding to the peak cylinder pressure gradually advanced, even at the MBT ignition timings. In addition, with the increase in φ(𝐻2), the cylinder pressure curve became steeper, i.e., the combustion rate and heat release rate in the cylinder became faster. The reasons for the above results are as follows. First, hydrogen direct injection forms a partial hydrogen-rich layered mixture near the spark plug and hydrogen has a low ignition energy, which together ensure the reliability of ignition and also lay a good foundation for the further propagation and development of flame. Second, hydrogen, as a gas fuel, does not need evaporation and atomization and has a rapid diffusion speed, which promotes the formation of a uniform mixture in the cylinder. Third, hydrogen has a fast flame propagation speed, which is conducive to the growth and development of the flame and promotes the propagation of turbulent flame and the combustion speed. Fourth, the quenching distance of hydrogen is short. Hydrogen addition can make the flame front spread closer to the cylinder wall, increase the contact area between the mixture and the flame front and promote combustion. It is worth noting that the improvement in the cycle-by-cycle variation of cylinder pressure was more obvious with a small φ(𝐻2) at λ = 1.0. With the further increase in φ(𝐻2), the improvement decreased. According to the above analysis, a small amount of hydrogen can enhance the reliability of ignition, increase the flame propagation speed, improve combustion process and reduce the cycle-by-cycle variation of cylinder pressure. The sensitivity of the cycle-by-cycle variation to φ(𝐻2) decreases. In addition, we can also see that when the φ(𝐻2) reached 20% at λ = 1.3, the cylinder pressure and the cycle-by-cycle variation of cylinder pressure increased more significantly compared to stoichiometric conditions. This is because the excellent fuel characteristics of hydrogen make up for the problems of n-butanol engines, such as difficult ignition, slow flame propagation speed and poor combustion stability under lean burn conditions, and significantly improve the in-cylinder combustion. In addition, a larger φ(𝐻2) was needed to obtain the best performance of the n-butanol engine because of the poor combustion environment under lean burn conditions.

**Figure 9:**
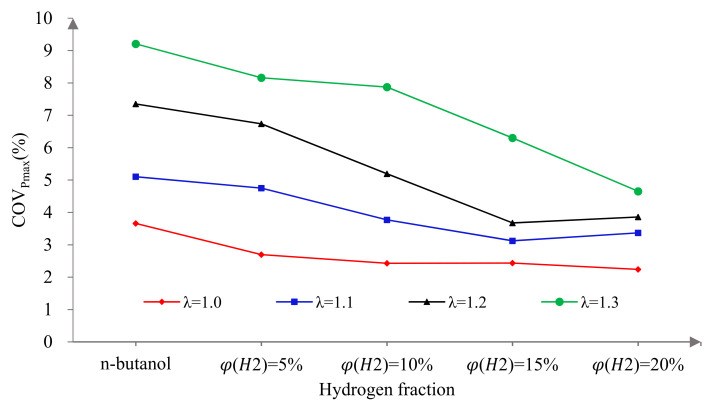
The effects of hydrogen fractions on COV_Pmax_ at different excess air ratios.

Figure 9 presents the effects of hydrogen fractions on the coefficient of the variation of Pmax (COVPmax) at four different excess air ratios. As can be seen from the figure, the larger the excess air coefficient, the larger the COVPmax. This is mainly because the greater the λ, the thinner the mixture in the cylinder, the more difficult the ignition and the slower the combustion rate, resulting in a reduction in the combustion isovolume and the increase in COVPmax. While, with the increase in φ(𝐻2), the COVPmax decreased gradually. This is because hydrogen has the advantages of a low ignition energy and fast flame propagation speed, which effectively ensure the stability of ignition, improve the combustion in cylinder, enhance the combustion stability and reduce the COVPmax. In addition, the effect of the hydrogen addition on reducing the COVPmax was more obvious under lean burn conditions. This is because misfires and incomplete combustion under lean burn conditions exacerbate the cycle-by-cycle variation. Hydrogen addition could promote the combustion in the cylinder, reduce the misfire rate and promote more complete combustion, thereby reducing the COVPmax.

**Figure 10:**
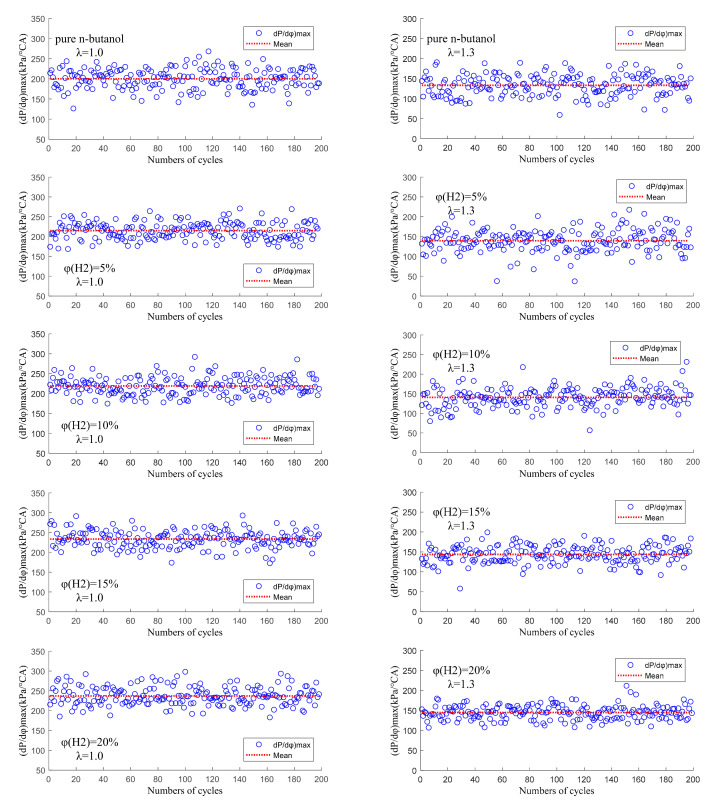
The cycle-by-cycle variation of the maximum rate of pressure rise ((dP/dφ)max) at different hydrogen fractions under λ = 1.0 and 1.3.

Figure 10 shows the cycle-by-cycle variation of the maximum rate of pressure rise ((dP/dφ)max) at different hydrogen fractions under λ = 1.0 and 1.3. Firstly, it can be seen from Figure 10 that the (dP/dφ)max of λ = 1.3 was significantly lower than that of λ = 1.0. This is mainly due to the thinner mixture and the low total energy per cycle under lean burn conditions. Secondly, with the increase in φ(𝐻2), the distribution of (dP/dφ)max became more and more concentrated and the hydrogen addition could effectively improve the value of the (dP/dφ)max. With the increase in φ(𝐻2), the (dP/dφ)max gradually increased. At λ = 1.0, the mean values of the (dP/dφ)max were 200.26 kPa/ CA, 214.84 kPa/ CA, 219.34 kPa/CA, 233.51 kPa/CA and 236.92 kPa/CA at the different φ(𝐻2), respectively. At λ = 1.3, the mean values of the (dP/dφ)max were 133.36 kPa/°CA, 139.51 kPa/°CA, 140.81 kPa/°CA, 143.80 kPa/°CA and 144.54 kPa/°CA at the different φ(𝐻2), respectively. The (dP/dφ)max was also still lower than the upper limit of engineering experience. Therefore, hydrogen addition can effectively improve engine stability and economy.

**Figure 11:**
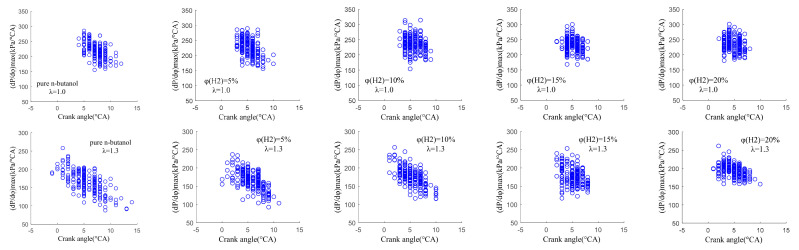
The correlation between the (dP/dφ)max and its corresponding crank angle at different hydrogen fractions under λ = 1.0 and 1.3.

Figure 11 shows the correlation between the (dP/dφ)max and its corresponding crank angle at the different hydrogen fractions under λ = 1.0 and 1.3. It can be seen from Figure 10 that the (dP/dφ)max obtained under lean burn conditions was significantly smaller than that obtained under stoichiometric conditions and that the corresponding crank angle was farther from TDC and more dispersed. This is mainly because, under lean burn conditions, the mixture in the cylinder is thin, the combustion speed is slow and the combustion duration is long, resulting in a delay in the combustion phase corresponding to the (dP/dφ)max. With the increase in φ(𝐻2), the (dP/dφ)max increased gradually, its corresponding crank angle was closer to TDC and the distribution range was more concentrated. The effects of mixture distribution, flow fields distribution and turbulence flow that interact violently on combustion in the cylinder were effectively reduced due to the fast diffusion velocity and flame propagation velocity of hydrogen. Therefore, the combustion speed in the cylinder is accelerated and the combustion duration is shortened after blending in hydrogen, which makes the distribution of (dP/dφ)max more concentrated and moves its corresponding crank angle forward.

#### 3.3.2. Cycle-by-Cycle Variation of Indicated Mean Effective

**Figure 12:**
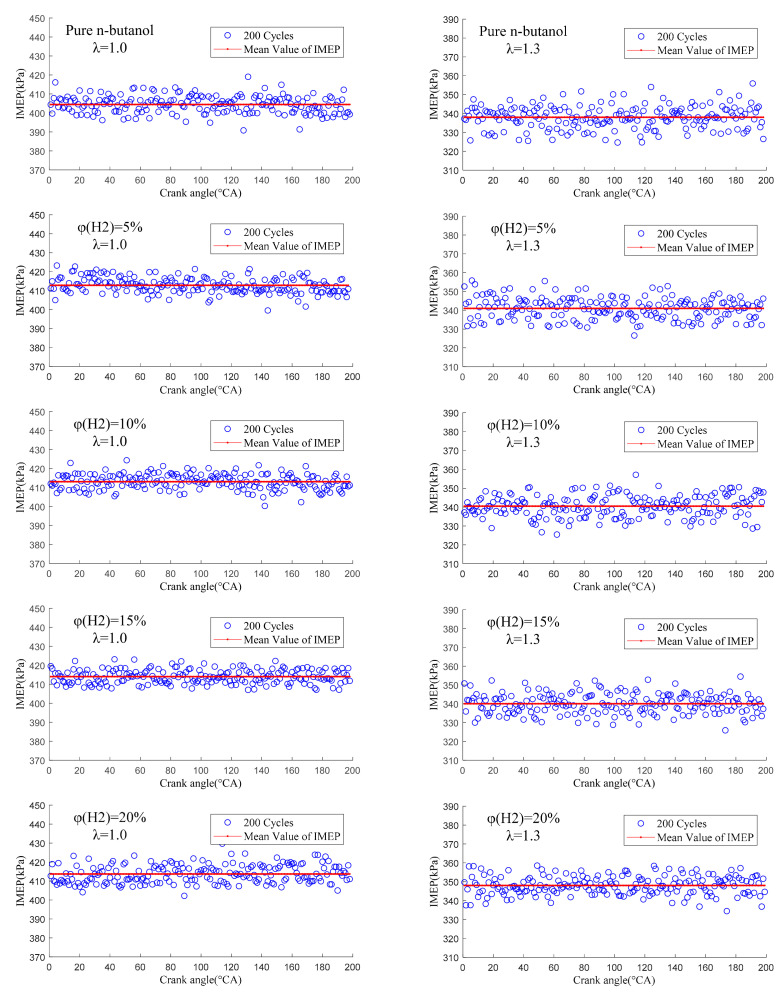
The cycle-by-cycle variation of IMEP at different hydrogen fractions under λ = 1.0 and 1.3.

Figure 12 displays the cycle-by-cycle variation of the IMEP at different hydrogen fractions under λ = 1.0 and 1.3. It can be seen that after blending in hydrogen, the average value of the IMEP increased, the fluctuation between each cycle became smaller and the distribution of IMEP was more concentrated and closer to the mean value. Under lean burn conditions, pure n-butanol engines have the problems of difficult ignition, long ignition delay time and slow flame diffusion speed, which makes the combustion incomplete and so, the cycle-by-cycle variation is serious. Hydrogen has a low ignition energy, fast flame propagation speed and wide ignition limit, which can effectively make up for the shortcomings of n-butanol fuel characteristics. Moreover, the composite injection mode of hydrogen direct injection forms a local hydrogen-rich region near the spark plug, which ensures the stability of ignition and improves the flame propagation speed. This makes the combustion process more stable and sufficient, reduces the frequency of irregular combustion and reduces the cycle-by-cycle variation of the IMEP. Further, when λ was 1.0, we can observe that with the increase in φ(𝐻2), the IMEP increased and the cycle-by-cycle variation became smaller, but the improvement effect showed a gradual weakening trend. This is because, in stoichiometric mode, the fuel is sufficient and the improvement in combustion by adding hydrogen mainly reflects the improvement in butanol engine ignition stability and initial fire core formation, which can be achieved by a small amount of hydrogen. The improvement of the IMEP and its cycle-by-cycle variation mainly depends on whether hydrogen is doped or not and is not sensitive to the amount of hydrogen.

In addition, when λ = 1.3, the value of the IMEP decreased and the cycle-by-cycle variation of the IMEP increased compared to λ = 1.0. When λ = 1.3, the cyclic distribution of the IMEP was improved more significantly after hydrogen addition and a larger hydrogen fraction ratio (20%) was required, which was different from under stoichiometric conditions. This is because the excellent fuel characteristics of hydrogen make up for the problems of n-butanol engines, such as difficult ignition, slow flame propagation speed and poor combustion stability under lean burn conditions, and significantly improve in-cylinder combustion. In addition, a larger φ(𝐻2) was needed to obtain the best performance of the n-butanol engine because of the poor combustion environment under lean burn conditions. As can be seen from the above analysis on Figure 8, this is mainly due to the wide ignition limit of hydrogen and its variety of excellent combustion characteristics, which were brought into better play under lean burn conditions.

**Figure 13:**
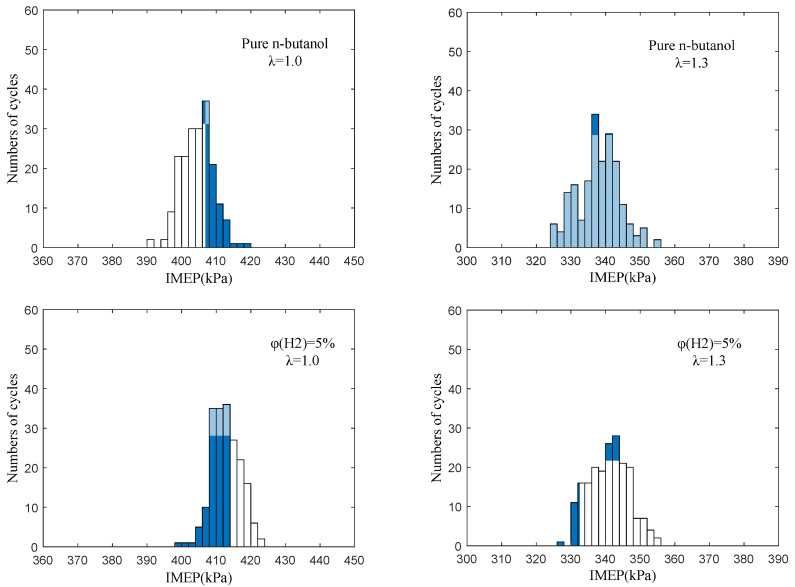

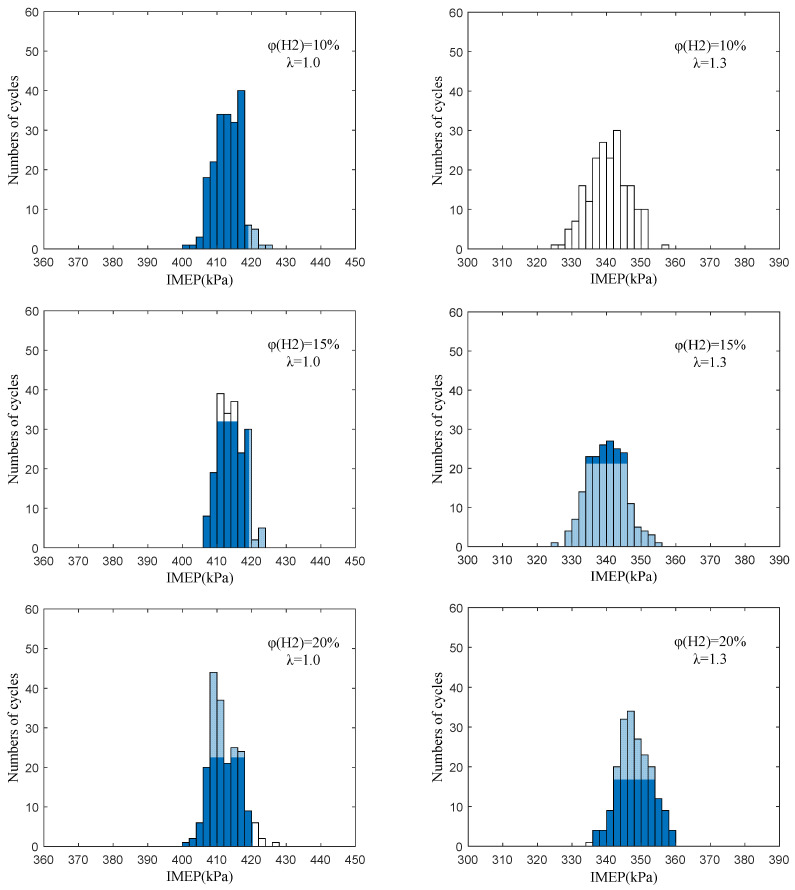
The distribution of IMEP at different hydrogen fractions under λ = 1.0 and 1.3.

The distribution of the IMEP at different hydrogen fractions under λ = 1.0 and 1.3 is presented in Figure 13. As can be seen, the distribution of IMEP without hydrogen addition covered a wide range, while the distribution of IMEP became more concentrated with the increase in φ(𝐻2). This is due to the low laminar flame velocity and ignition energy of hydrogen, which can effectively improve ignition and promote flame propagation, thus improving the combustion process. In addition, compared to λ = 1.0, the value of the IMEP under lean burn conditions decreased, the whole data of IMEP scattered over a wide range and more data values appeared in the region with the large or small IMEP. This is mainly because, under lean burn conditions, the concentration of the mixture in the cylinder is low and the flame development speed decreases, which affects the development and growth of the initial fire core. Therefore, the combustion in the cylinder is greatly affected by the flow field and turbulence distribution in the cylinder, so the cycle-by-cycle variation in IMEP is obvious. Therefore, hydrogen addition can improve the combustion stability and effectively improve the lean burn capacity of an n-butanol engine.

**Figure 14:**
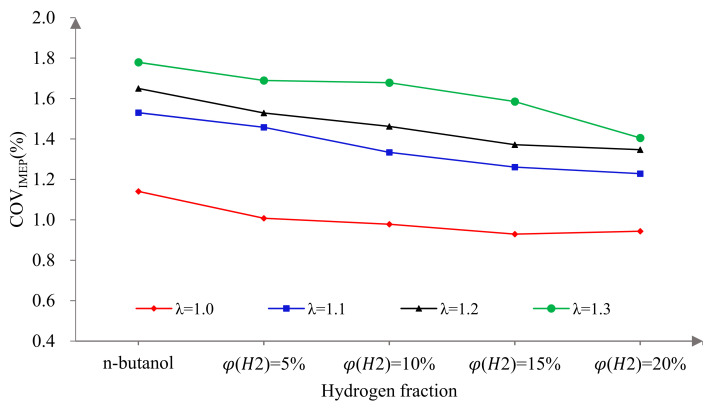
The effects of hydrogen fractions on COV_IMEP_ at different excess air ratios.

Figure 14 shows COV_IMEP_ versus excess air ratio at different hydrogen fractions. As can be seen, COV_IMEP_ increased gradually with the increase in λ. This is mainly because with the increase in λ, the mixture in the cylinder becomes thinner, which makes ignition difficult and the flame propagation speed slow. These lead to a slow combustion speed and combustion deterioration and, finally, aggravate the cycle-by-cycle variation of the engine. When λ was 1.0, the COV_IMEP_ decreased sharply at first and then tended to be flat with the increase in φ(𝐻2). It is worth noting that when φ(𝐻2) was 5%, the physicochemical properties of hydrogen as an auxiliary fuel were better reflected and the improvement effect on COV_IMEP_ was the most significant. This is because a small amount of hydrogen is able to achieve the purpose of ignition and support combustion. Therefore, the reduction in COV_IMEP_ mainly depends on whether hydrogen is mixed or not and is not sensitive to the hydrogen fraction. When λ was more than 1.0, the COV_IMEP_ decreased gradually with the increase in φ(𝐻2). This is because the in-cylinder mixture could achieve stable ignition at λ = 1.0. With the further increase in λ, misfires and incomplete combustion occurred and the cycle-by-cycle variation was intensified. Hydrogen addition can accelerate the combustion speed because of its low ignition energy, fast flame propagation speed and wide ignition limit, which can reduce misfires and incomplete combustion in the cylinder and effectively reduce cycle-by-cycle variation and improve combustion stability. Therefore, hydrogen addition can effectively reduce COV_IMEP_ and the lean burn performance of an n-butanol engine. However, compared to a = 1, the lean burn line needed a higher hydrogen ratio to obtain a better effect under lean burn conditions.

## 4. Conclusions

An experimental study on cycle-by-cycle variation was conducted on an n-butanol SI engine with hydrogen direct injection under lean burn conditions. The purpose of this study is to improve the power performance and stability of n-butanol engine by hydrogen direct injection The following conclusions could be drawn based on the experimental results:

(1) The IMEP increased with the increase in φ(𝐻2) under any value of λ. When λ = 1.0, the IMEP improved most significantly with a 5% hydrogen fraction and was not sensitive to φ(𝐻2). When λ was larger than 1.0, a larger φ(𝐻2) could effectively improve the IMEP;

(2) Hydrogen direct injection could effectively improve the combustion performance of n-butanol engines. The θ0-10 and θ10-90 all decreased gradually with the increase in φ(𝐻2). Under λ = 1.1, 1.2 and 1.3, the θ0–10 values were lower than the original engine level at λ = 1.0 when the φ(𝐻2) was 20%;

(3) Hydrogen addition could significantly improve the cycle-by-cycle variation of cylinder pressure, especially under lean burn conditions. When λ = 1.3, a 20% hydrogen fraction significantly reduced the cycle-by-cycle variation of cylinder pressure and the probability of misfire;

(4) The Pmax and (dP/dφ)max increased and their cycle-by-cycle variations decreased by blending in hydrogen. The correlation between the (dP/dφ)max and its corresponding crank angle also became weaker with the increase in λ, while they tended to be strongly correlated with the increase in φ(𝐻2);

(5) The coefficient of variation of the Pmax and the IMEP decreased with the increase in λ, while they decreased obviously after blending in hydrogen. Furthermore, a 5% hydrogen fraction improved their cycle-by-cycle variations most significantly at λ = 1.0 and a larger hydrogen fraction was needed under lean burn conditions;

(6) In conclusion, hydrogen direct injection can improve n-butanol engine performance, enhance stable combustion and reduce cycle-by-cycle variation. With the increase in the excess air coefficient, a higher hydrogen ratio is needed to effectively improve the combustion and cycle variation performance of butanol engines. Exploring the influence of hydrogen direct injection on butanol engine emissions will be the focus of the next step of this work in order to meet more stringent emissions regulations in the future.

## Figures and Tables

**Table 1:** The properties of alcohol fuels.

	Methanol	Ethanol	Butanol	Gasoline	Hydrogen
Molecular formula	CH_3_OH	C_2_H_5_OH	C_4_H_9_OH	C_5_–C_12_	H_2_
Cetane number	3	8	25	5–25	-
Research octane number	111	108	96	80–99	-
Density, g·L^−1^	792 ^c^	789 ^c^	808 ^c^	720–780 ^c^	0.089 ^a,b^
Viscosity (Pa·s) at 20 °C	0.61	1.2	3.64	0.28–0.59	-
Lower caloric value, MJ·kg^−1^	19.9	26.8	33.1	42.7	119.7
Latent heat of evaporation, MJ·kg^−^^1^ (25 °C)	1.11	0.9	0.58	0.38–0.50	-
Saturated vapor pressure, kPa (38 °C)	31.69	13.8	2.27	31.01	-
Stoichiometric air–fuel ratio	6.49	9.02	11.21	14.7	34.5
Flammability limits (Vol%)	6.0–36.5	4.3–19.0	1.4–11.2	0.6–8.0	4.0–76.0
Oxygen content (Mass%)	50	34.8	21.6	-	-
Laminar flame speed, cm·s^−1^ (25 °C)	68	63–	48–53	37–43	185
Autoignition temperature, °C	470	434	385	~300	585
Minimum ignition energy, mJ	0.215	0.63–	-	0.24	0.02

^a^ at 1 bar; ^b^ at 273 K; ^c^ at 20 °C.

**Table 2:** The main parameters of the test engine.

Engine Parameter	Parameter Values
Engine Type	four cylinders; dual injection; spark-ignited
Compression ratio	9.6:1
Bore × Stroke, mm	82.5 × 92.8
Displaced volume, L	1.984
Maximum power, kW	132 (5000–6000 rpm)
Maximum torque, N·m	320 (1600–4000 rpm)

**Table 3:** The main test equipment of the experiment.

Parameter	Manufacturer	Range	Precision	Production Type
Speed	Luoyang Nanfeng Electromechanic Equipment Manufacturing Co., Ltd.	0~6000 rpm	≤±1 r/min	CW160
Torque	Luoyang Nanfeng Electromechanic Equipment Manufacturing Co., Ltd.	0~600 N·m	≤±0.28 N·m	CW160
Excess air ratio	ETAS Engineering TOOLS	0.700~32.767	≤±1.5%	LAMBDA LA4
N-butanol mass flow meter	ONO SOKKI (Onokazu detector)	0.2~82 kg/h	±0.01 g/s	Ono Sokki DF-2420
Hydrogen mass flow meter	Beijing SINCERITY	0.2~1 kg/h	±0.2%	DMF-1-1AB
Crank angle	Kistler Instrument China Ltd.	0~720°	≤±0.5°	Kistler-2614B4
Cylinder pressure	DEWETRON GmbH.	0~20 MPa	≤±0.5%	AVL-GU13Z-24

## Data Availability

Not applicable.

## Abbreviations

**Abbreviation**	**Meaning**
SI	Spark ignition
MAP	Intake manifold absolute pressure
λ	Excess air ratio
φ(𝐻2)	Hydrogen fraction
MBT	Minimum ignition advance for best torque
TDC	Top dead center
ATDC	After top dead center
BTDC	Before top dead center
IMEP	Indicated mean effective pressure
Pmax	Peak in-cylinder pressure
APmax	Position of Pmax
(dP/dφ)max	Maximum rate of pressure rise
θ0–10	Flame development duration
θ10–90	Rapid combustion duration
COV	Coefficient of variance
